# Sensor Fault Diagnostics Using Physics-Informed Transfer Learning Framework

**DOI:** 10.3390/s22082913

**Published:** 2022-04-11

**Authors:** Furkan Guc, Yangquan Chen

**Affiliations:** Department of Mechanical Engineering, University of California Merced, Merced, CA 95343, USA; fguc@ucmerced.edu

**Keywords:** data-driven approaches, dynamic mode decomposition with control, fault diagnostics, transfer learning

## Abstract

The field of smart health monitoring, intelligent fault detection and diagnosis is expanding dramatically in order to maintain successful operation in many engineering applications. Considering possible fault scenarios that can occur in a system, indicating the type of fault in a sensor is one of the most important and challenging problems in the area of intelligent sensor fault diagnostics. Within this frame of reference, we extended the physics-informed transfer learning framework, first presented previously for a fault cause assignment, to the level of sensor fault diagnostics for a range of different fault scenarios. Hence, the framework is utilized to perform intelligent sensor fault diagnostics for the first time. The underlying dynamics of the reference system are extracted using a completely data-driven methodology and dynamic mode decomposition with control (DMDc) in order to generate time-frequency illustrations of each sample with continuous wavelet transform (CWT). Then, sensor fault diagnostics for bias, drift over time, sine disturbance and increased noise sensor fault scenarios are achieved using the idea of transfer learning with a pre-trained image classification algorithm. The classification results yields a good performance on sensor fault diagnostics with 91.5% training and 84.7% test accuracy along with a fair robustness level with a set of reference benchmark system parameters.

## 1. Introduction

The demand and utilization of fault diagnosis and health monitoring tools are increasing dramatically over the last decade. A variety of applications in this context evolved into recognized instruments in many areas of engineering to preserve a successful operation in a variety of applications. As one of the key components in complex applications, sensors are open to introduce many different faults into the system due to their nature and working environment [1]. The main problem in the health monitoring and fault diagnosis framework can be considered as the identification procedure of the sensor fault type, which is aroused during the system operation. Although some of the sensor fault types are apparent, such as catastrophic failures, lots of sensor fault cases, namely bias, drift, low-frequency oscillations and increased sensor noise, are hard to distinguish during the systems’ operation. Generally, only the input and output data are available to perform health monitoring without additional specialized sensors. In this context, the fault diagnosis framework became a much more complicated and challenging problem along with the hidden physical characteristics of the fault type.

There are a variety of applications in the literature focused on the sensor fault diagnosis framework. A general methodology for fault diagnosis with the emphasis on the isolation of multiple sensor faults is presented in [2]. A comprehensive review on fault diagnosis methods with the current state-of-the-art in the field is presented in [1]. An online drift compensation estimation along with applications of Kalman filters introduced in [3] and the spatial kriging methodology for sensor drift detection are presented in [4]. Then, a sensor drift detection technique is introduced in [5] with the aid of discrete wavelet transform (DWT) and with the aid of grey models. As an emerging and handy tool on many engineering applications, the digital twin framework is utilized in [6] for autonomous maintenance and fault detection. Comprehensive methodology for a ball screw degradation prognosability is presented in [7]. A model-based approach for the electric vehicle battery sensor fault diagnosis problem is introduced in [8]. These contributions handle the sensor fault diagnosis problem in many different aspects and introduce valuable methods with high accuracy sensor fault diagnosis. The fundamental limitation of these methods is that they require an additional sensor, specialized sensor placement and a prior physics or system model. However, only the input and output measurements from the system are available to perform effective fault diagnosis most of the time.

As they became standardized in many engineering operations, data-driven techniques are utilized in the fault diagnosis context in a variety of different applications, such as aircraft engines [9], induction motor drive systems [10] and many other engineering aspects [11,12,13]. On the other hand, machine learning techniques are also highly exploited in the framework of fault diagnostics and health monitoring with neural networks [14,15,16], ensemble learning [17], unsupervised learning [18], convolutional neural networks [19], extremely randomized trees [20], ANN-assisted sensor information fusion [21] and generative adversarial networks [22]. The utilization of machine learning algorithms can dramatically increase the sensor fault diagnosis. As one of the most critical drawbacks, they require a big volume of sample sets along with high-dimensional system data and computationally heavy training procedures [23].

The fundamental motivation of using a data-driven approach is to eliminate the requirement of a system model or prior information regarding underlying system dynamics. However, handling this high-volume system data can also be considered as one of the challenges in fault diagnosis frameworks [24]. With the increasing availability of big data, practical engineering applications with control methodologies utilize dimensionality reduction techniques in order to handle high-dimensional system data. These methods enable users to create low-dimensional sub-spaces of complex system dynamics [25,26,27]. In this context, dynamic mode decomposition (DMD) is a completely data-driven, emerging method used in order to achieve linear reduced-order system dynamics. The equation-free methodology allow the user to obtain the underlying physics of the corresponding system without the need for any prior knowledge for a system model. It is first utilized to understand the complex fluid dynamics [28,29], then extended to the dynamics community [24,30]. It is one of the most critical extensions to the DMD methodology included the control aspect of the dynamic system. Dynamic mode decomposition with control (DMDc) is introduced in [31] for systems with an input signal. Moreover, sensor fault diagnosis methodologies also exploited DMD methodology in rolling bearings [32] and flight test data [33].

In this paper, a systematic methodology is applied to achieve sensor fault diagnosis using a purely data-driven dimensionality reduction technique, DMDc and idea of transfer learning. With the aid of these, our proposed methodology overcomes the requirement of prior system knowledge and the burden of handling a high-volume of data with a from-scratch training process. Over the last decade, the fault diagnosis and health monitoring framework became one of the most critical aspects of complex systems. However, identifying the exact fault in a sensor using only the system measurements is still a critical process in the literature with a growing research impact [1]. This process can be elevated by utilizing the given underlying dynamics or physics of the system, which are defined as physics-informed learning [34,35,36]. The framework is first introduced in [37], in order to perform a fault cause assignment within a system, namely with the actuator and sensor. Then, the proposed framework is extended to the sensor fault diagnosis in order to classify different types of faults that can be occurred in the sensor. The introduced methodology utilizes the underlying system dynamics obtained by completely data-driven tools in the process of classification. To achieve the sensor fault diagnosis, only the system input–output measurements are utilized without the requirement of system model. The underlying physics of the system obtained through DMDc are transformed into time-frequency illustration images with the help of the continuous wavelet transform (CWT). Finally, generated labelled images are utilized in the classification model to achieve fault diagnosis. In this context, a previously trained model for image classification, *GoogLeNet* [38] is used to decrease the training time with the idea of transfer learning. As the procedure still utilizes a data-driven approach, it shares the high-volume labelled system data requirement of supervised learning algorithms, although dimensionality reduction techniques are utilized to extract underlying dynamics. With the aid of the systematic framework proposed for sensor fault diagnosis, a good performance is achieved for both training and test data sets together with a robustness study for a set of reference benchmark system parameters.

The structure of the paper is presented as follows: Section 2 provides the introductory information and required methodology for the step-by-step explanation performed in the physics-informed transfer learning framework. The preliminaries of the case study are presented in Section 2.1, the concept of dynamic mode decomposition with control in Section 2.2, time-frequency illustrations along with CWT in Section 2.3 and transfer learning image classification in Section 2.4. Then, Section 3 presents fault diagnosis results for the presented case study for a variety of sensor faults. Finally, Section 4 discusses the final comments on the utilization of the framework for the sensor fault diagnosis.

## 2. Methodology

**Figure 1:**
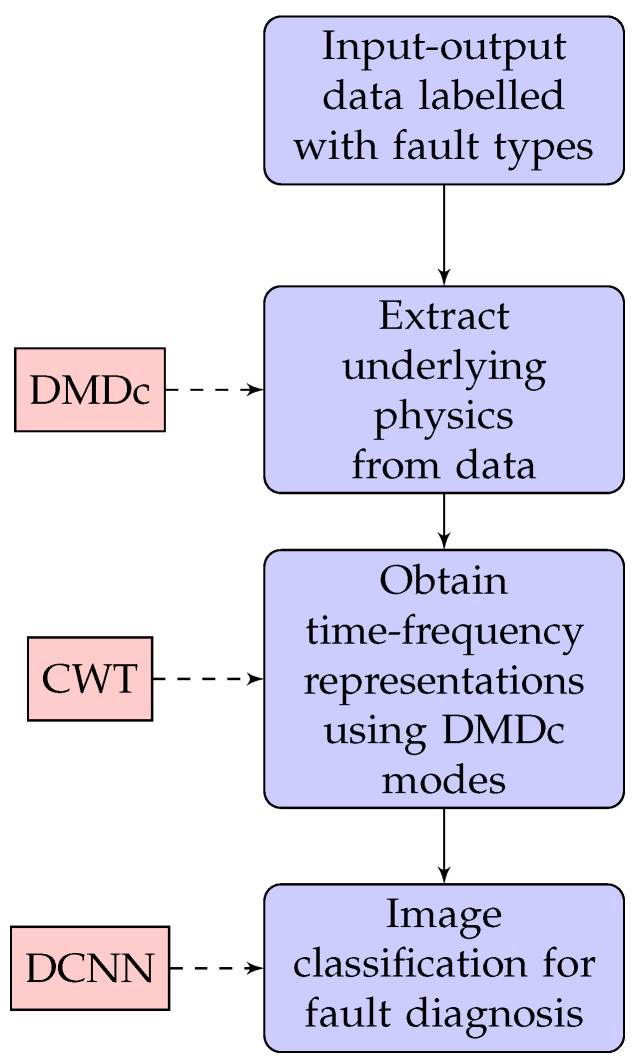
The flowchart of the extended sensor fault diagnosis process based on the physics-informed transfer learning framework proposed in [37].

The overall methodology and general framework of the sensor fault diagnosis with physics-informed transfer learning is presented in this section. The general structure of the methodology is first introduced in [37] and the extension of the methodology for the sensor fault diagnosis is demonstrated in Figure 1. The labelled input–output data streams from the system measurements are generated using a case study simulations. Then, the underlying system dynamics are extracted from the data using DMDc, and time-frequency image representations of DMDc modes are generated using CWT. In the final step, fault diagnosis are performed using the image classification model based on deep convolutional neural networks (DCNN). A similar process is utilized in [37] to assign the same fault introduced by an actuator or sensor.

### 2.1. Reference Case Study

**Figure 2:**
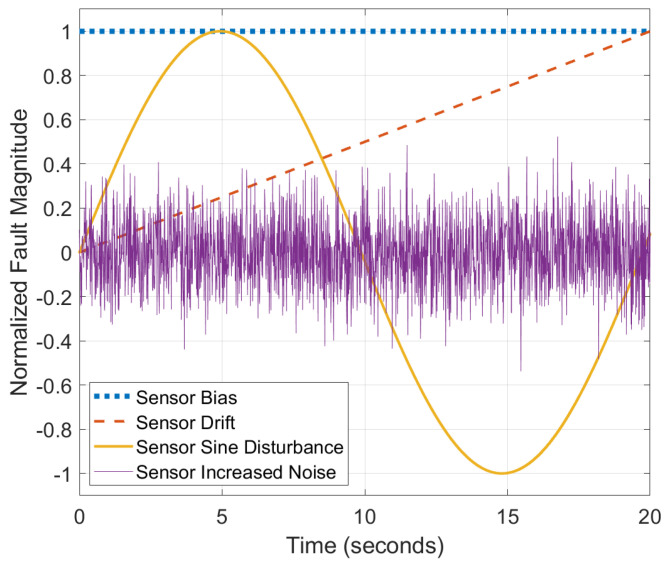
Normalized sample sensor fault scenarios over time.

An example case study is introduced in order to investigate sensor fault diagnosis on a generalized process control example. Five scenarios for the injected fault types are defined as nominal, bias, drift over time, sine disturbance and increased noise. Normalized sample sensor fault scenarios are illustrated in Figure 2.

**Figure 3:**
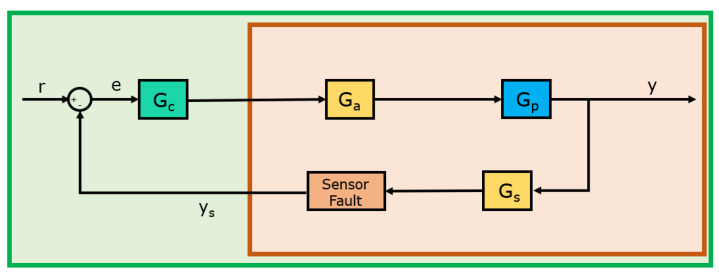
Block diagram of the generalized case study.

A generalized reference control system is given in Figure 3. As we consider only sensor fault diagnosis in this context, other possible fault sources are eliminated. Therefore, the actuator transfer function $G_{a}$ and sensor transfer function $G_{s}$ are selected to be 1. Moreover, sensor disturbance $d_{a}$ is considered as 0. Moreover, a PID controller $G_{c}$ is implemented to complete the feedback structure. Finally, the plant transfer function $G_{p}$ is introduced as one of the most commonly utilized control benchmark, the first-order plant with time delay (FOPTD). Over 80% of temperature systems with control implementations in engineering applications can be modeled on this structure. The FOPTD system is normalized with respect to two parameters:

(1) $$G_{p}=\frac{1}{Ts+1}e^{-(L/T)Ts}=\frac{1}{s^{\prime }+1}e^{-L^{\prime }s^{\prime }}$$

with $s^{\prime }=Ts$, and $L^{\prime }=L/T$ which enables one to eliminate normalized parameters for the plant gain ($K^{\prime }$) and plant time constant ($T^{\prime }$) is 1. Using this structure, the number of parameters in FOPTD model is decreased to one instead of three. The only parameter in the normalized form is the normalized delay $L^{\prime }$. The main scenario in the case study is considered as $L^{\prime }=1$. Further discussion on the robustness of the proposed methodology is constructed using two variations of the normalized delay parameter, corresponding $L^{\prime }=0.1$ and $L^{\prime }=10$.

**Figure 4:**
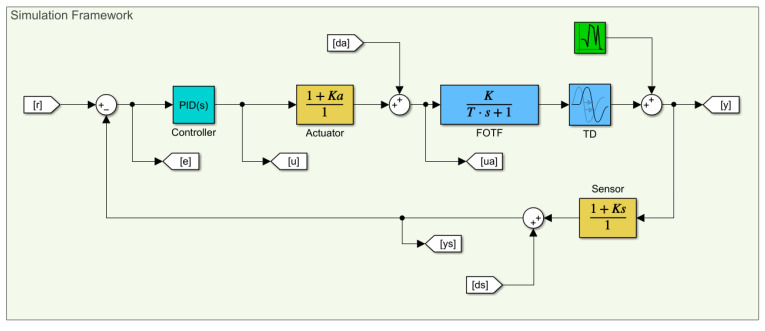
Simulation framework created in Simulink.

One of the key limitations in the framework of the fault diagnosis is the fact that the system measurements are restricted to only the reference *r* and the sensor feedback $y_{s}$ signals. Therefore, from the point of sensor fault diagnosis, the overall structure can be considered as a black box configuration. The green box in Figure 3 represents the black box model, which means only the signals outside of the green box are available to utilize. In order to generate train and test data sets that include all possible scenarios, a system simulation is created in the MATLAB/Simulink environment and given in Figure 4. Each sample in the data sets is generated with a simulation environment with the aid of Gaussian noise, purposely injected to the simulation with different seeds. This enables one to generate each run with unique Gaussian noise. The noise level in the simulation is introduced as 10%, which corresponds to a 20 dB signal-to-noise ratio. The simulation time for each sample is considered as 20 s and the reference signal is introduced as a unit square wave with a period of 10 s. Finally, 300 unique runs are generated for each scenario in order to generate a rich data set including 1500 runs.

### 2.2. Dynamic Mode Decomposition with Control

The use of data-driven techniques in many engineering applications is dramatically increased over the last few decades. As a completely data-driven method, the dynamic mode decomposition (DMD) is utilized in various contexts to acquire linear reduced-order models of any dynamic system with input–output data. DMD does not require any previous knowledge on underlying physics between the input and output data. Only the revealed input and output measurements from the system are used in order to extract spatial temporal modes and patterns in the data [24,37].

Then, the DMD framework is extended for systems that include actuation, such as many different control applications. The dynamic mode decomposition with control (DMDc) includes system measurements together with the applied control signal in order to extract the governing dynamics of the system [31,37]. Then, DMDc became one of the powerful tools that enable control applications to use data-driven system dynamics in various engineering applications.

The mathematical roots of the DMDc framework start with the signals obtained from the system, namely the reference input and sensor measurements. Once the reference case study is considered, the reference *r* and sensor feedback $y_{s}$ signals are the only signals measured. DMDc assumes that system measurements have an approximately linear relation with respect to

(2) $$x_{k+1}\approx Ax_{k}+Br_{k},$$

where $x_{k}$ and $r_{k}$ are the windowed time domain data or snapshot of the sensor measurement and reference input signals, respectively. Then, $x_{k}$ and $r_{k}$ are reshaped into tall matrices. Each tall column in matrices refers to time-shifted snapshots from the sensor measurements or input signals of the system. In this context, sensor measurements can be represented as

(3) $$X=\left[\begin{array}{cccc} | & | & \; & |\\ x_{1} & x_{2} & \cdots & x_{m}\\ | & | & \; & | \end{array}\right]\;X^{\prime }=\left[\begin{array}{cccc} | & | & \; & |\\ x_{2} & x_{3} & \cdots & x_{m+1}\\ | & | & \; & | \end{array}\right]$$

where **X′** can be considered as the time-shifted copy of **X**. **X** consists of columns [1,m] and **X′** consists of columns [2,m+1]. Within the same concept, the reference input signal *r* signal is also reconstructed as a tall matrix consisting of snapshot columns as

(4) $$R=\left[\begin{array}{cccc} | & | & \; & |\\ r_{1} & r_{2} & \cdots & r_{m}\\ | & | & \; & | \end{array}\right].$$

Approximately linear dynamics of the system can be written in terms of tall data matrices defined above as:

(5) $$X^{\prime }\approx AX+BR.$$

**Figure 5:**
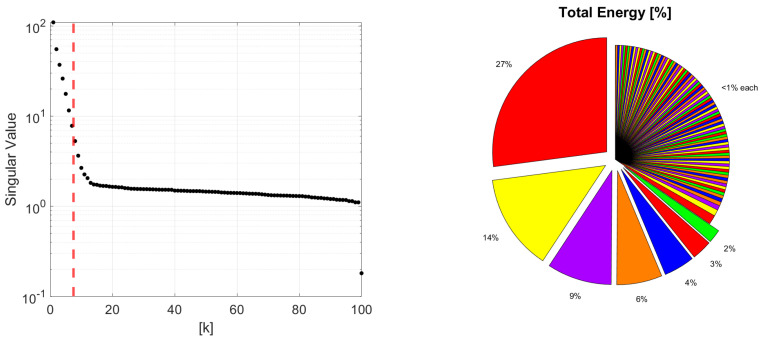
Singular values and cumulative energy contributions for a sample reference case, where the first 7 singular values contribute 65% of the total energy.

By the very nature of the DMDc methodology, the extracted linear reduced-order dynamics of the system can be considered as $\tilde{A}$ and $\tilde{B}$. These reduced-order models are the truncated version of the approximately linear dynamics with respect to singular value decomposition (SVD) [31]. To demonstrate the truncation process, a sample run from the reference case study is utilized. Singular values and cumulative energy contributions are presented for a sample reference case in Figure 5, where *k* represents each singular value sorted hierarchically.

**Figure 6:**
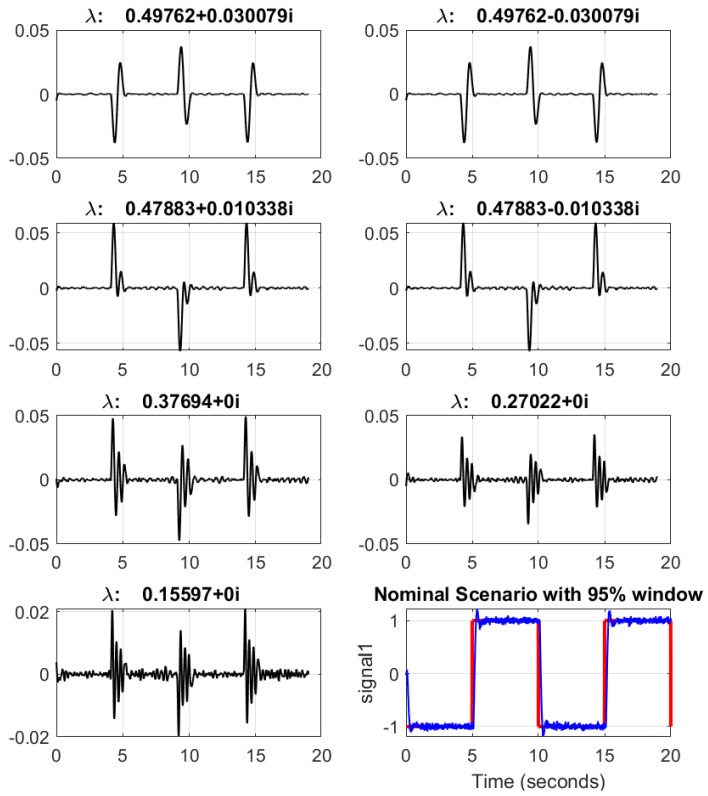
Data spatial temporal modes for nominal scenario in the reference case study using the first seven singular values.

**Figure 7:**
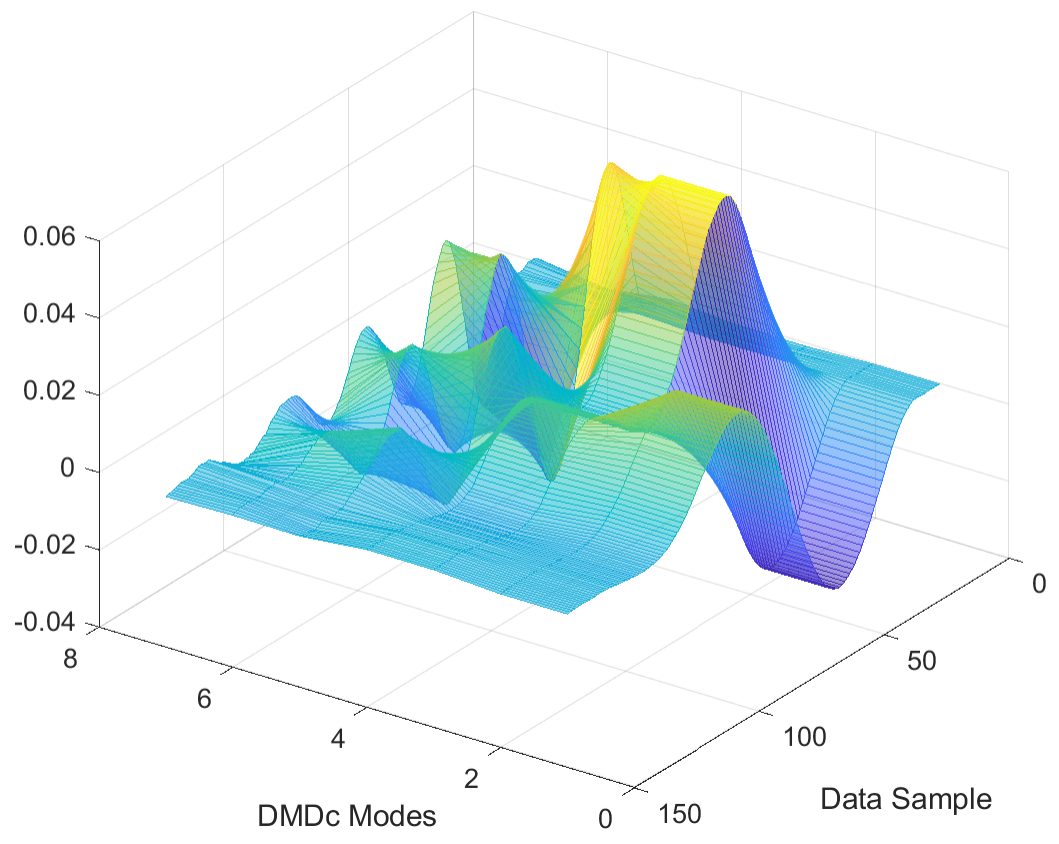
Combination of seven DMDc modes within one transient of the square reference tracking.

By using the shape of the total energy contribution graph, the truncation level is determined. As the slope of the total energy contribution graph is decreasing dramatically after k=7, the order is selected as 7 and highlighted with a red-dashed line in Figure 5. To investigate the selected number of DMDc modes, the corresponding 7 data modes obtained, with respect to their contribution of the total dynamics, are presented in Figure 6 for a sample nominal scenario. Furthermore, Figure 7 illustrates a combination of 7 DMDc modes within one transient of the square reference tracking.

### 2.3. Time-Frequency Representations Using CWT

**Figure 8:**
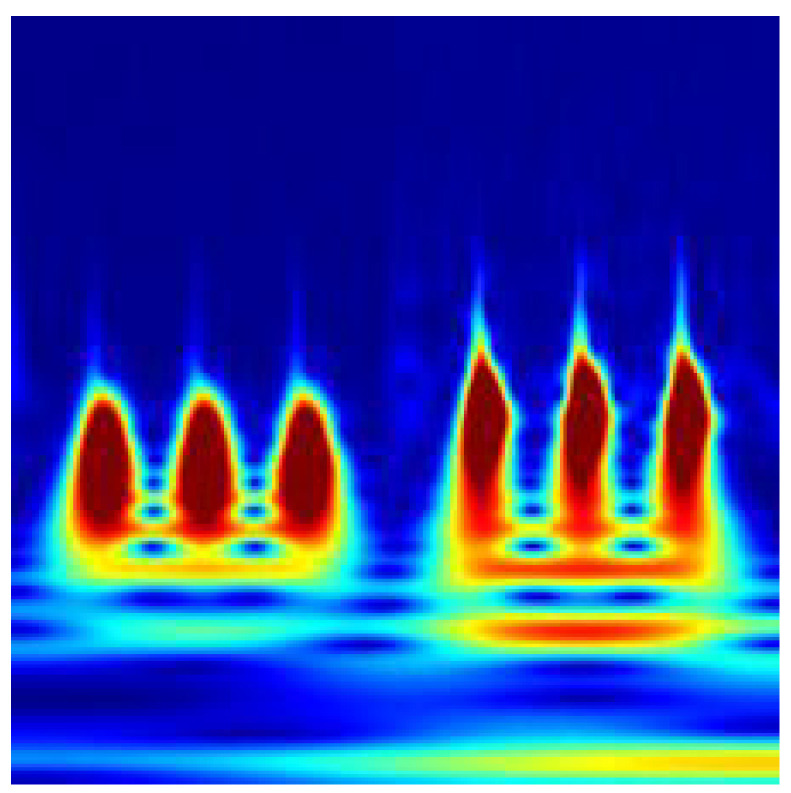
Time-frequency representation for the nominal scenario.

**Figure 9:**
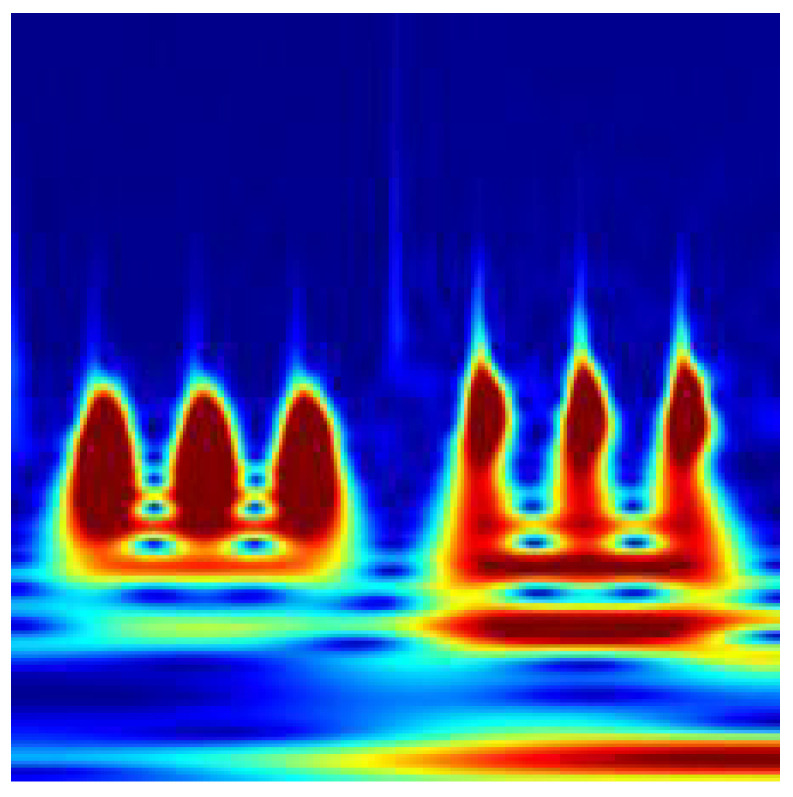
Time-frequency representation for the bias fault scenario.

**Figure 10:**
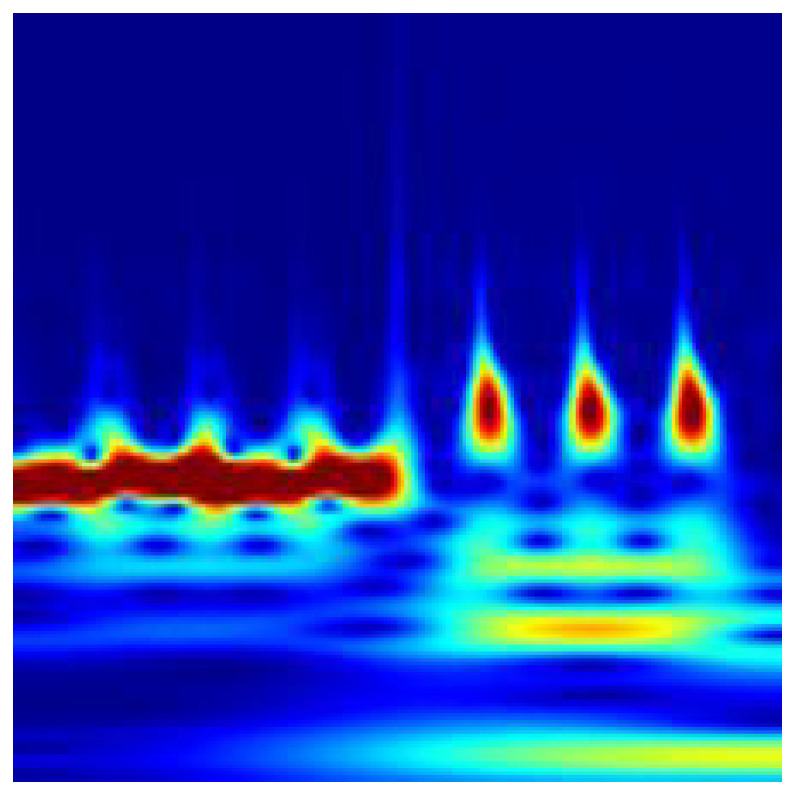
Time-frequency representation for the sine fault scenario.

In order to utilize the underlying physics of the corresponding system extracted using a completely data-driven method, DMDc, the dynamic modes of the first two dominant contributors are transformed into images of time-frequency representations. To perform the transformation of DMDc modes into images, there are many different time-frequency representations or illustrations, such as the Short-Time Fourier Transform (STFT) [39] and Wigner-Ville distribution (WVD) [40]. However, the continuous wavelet transform (CWT) was enabled to obtain more distinctive visual properties along with a successful performance in fault diagnosis using 2D representations of time domain signals shown in [19,22]. For each DMDc mode extracted from the reference case study with a different fault scenario, scalograms are obtained using CWT. These time-frequency representations correspond to the absolute values of CWT coefficients. To overcome a high-volume data set, a CWT filter bank is constructed and used for each case with the same parameters. Finally, 224 × 224 time-frequency representation images are obtained for each data in the generated set. Hence, the image set can be utilized as the default input layer for many image classification models. Sample images for the time-frequency representations are introduced in Figure 8 for the nominal scenario, in Figure 9 for the sensor bias fault scenario, and in Figure 10 for the sine disturbance fault scenario. The distinct features and differences for different fault scenarios can also be visually identified.

### 2.4. Image Classification Using Transfer Learning

The image set generated with the CWT can be considered as the input layer of the image classification models. To perform a sensor fault diagnosis, a deep convolutional neural network (DCNN) image classification structure is constructed. In this context, the images generated using DMDc modes represent the physics information to be utilized in the classification process. One of the key points in the implementation of the image classification with DCNN is the initiation of the model. Although different applications in deep learning implementations increases the performance dramatically, training a DCNN image classification model from scratch is still a computationally heavy task. Hence, one of the most commonly utilized and efficient ideas is to implement a transfer learning concept. In this context, previously trained DCNN models are used to improve the training performance and decrease the computational time.

**Figure 11:**
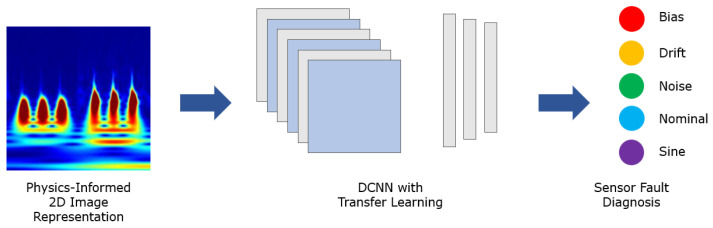
Workflow representation for DCNN transfer learning strategy.

Different alternative image classification algorithms in the ImageNet Large-Scale Visual Recognition Challenge (ILSVRC) are investigated, such as *AlexNet* introduced in [41] and the Residual Neural Network (*ResNet*) presented in [42]. In the reference case study, a well-established DCNN architecture for image classification, the *GoogLeNet* model is used [38], as it achieves similar error levels using fewer numbers of parameters. A rigorous comparison between deep neural network models is given in [43]. Furthermore, the application of a set of different DCNN algorithms for a practical application demonstrated that *GoogLeNet* performs better in terms of accuracy even if it has less parameters, and the most successful learning methodology is transfer learning [44]. In the *GoogLeNet* DCNN model, primary layers shall be constructed as basic filters for a universal feature detection such as colors or edges within the corresponding image. The earliest *GoogLeNet* model is trained for the image classification of 1000 different labels [38]. With the aid of the transfer learning strategy, the efficiency of the DCNN training process is dramatically increased [43,44]. The representation of the training and fault diagnosis workflow is illustrated in Figure 11.

As stated in the time-frequency representation process, the input layer of the *GoogLeNet* model consists of a 224 × 224 × 3 RGB image. The data set, including 1500 samples of five different scenarios, is separated in order to obtain three different data sets, namely, the training, validation and test. These data sets consist of 1200, 150 and 150 images, respectively, with random selection. The training data set consisting of 1200 samples is utilized in order to perform training operations. Then, the validation data set consisting of 150 samples is utilized once in each 10 epochs to check the validation accuracy during training operations. Then, the test data set consisting of 150 samples is utilized to check the test accuracy after the training process. Finally, the training process is completed for the transfer learning of the DCNN model using the Stochastic Gradient Descent with Momentum algorithm along with the DCNN model training parameters given in Table 1.

The training process is conducted on a laptop computer with an Intel Core i7-8750H CPU processor running at 2.20 GHz using 16 GB of RAM and GeForce GTX 1050 with Windows 11 and MATLAB r2021b. The gradient magnitude is utilized to track and terminate the training procedure. Within these configurations, the average training time for a sample set is 25 min using the MATLAB function *gputimeit*.

## 3. Results

**Figure 12:**
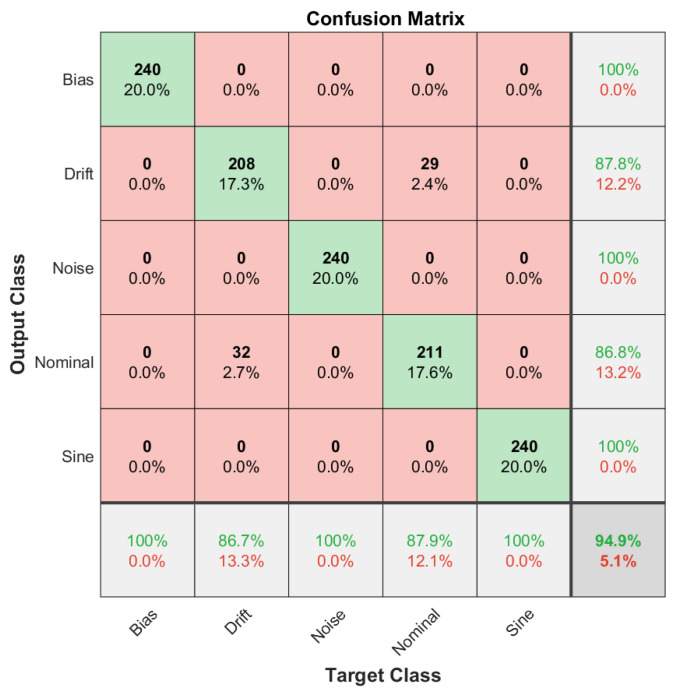
Confusion matrix for the training data set showing that a huge proportion of the misclassified samples are between drift fault and nominal scenarios.

**Figure 13:**
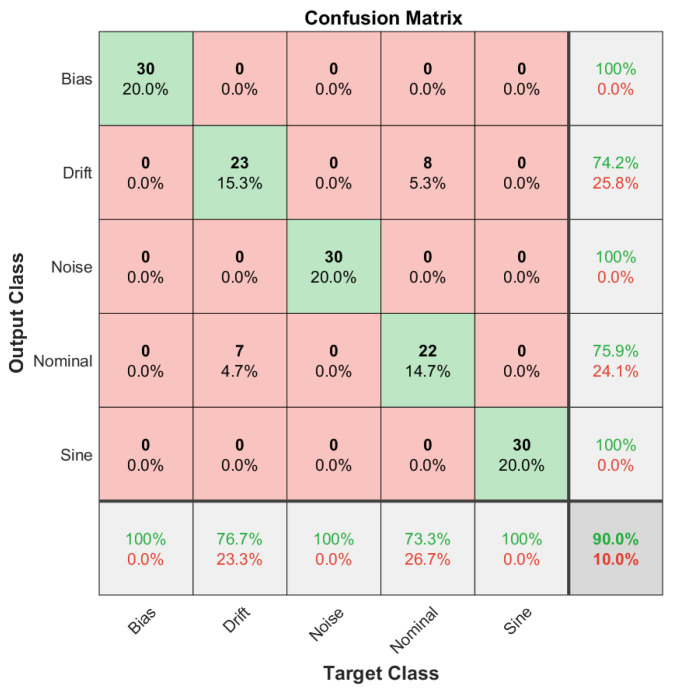
Confusion matrix for the validation data set showing that a considerable amount of the misclassified samples are between drift fault and nominal scenarios.

**Figure 14:**
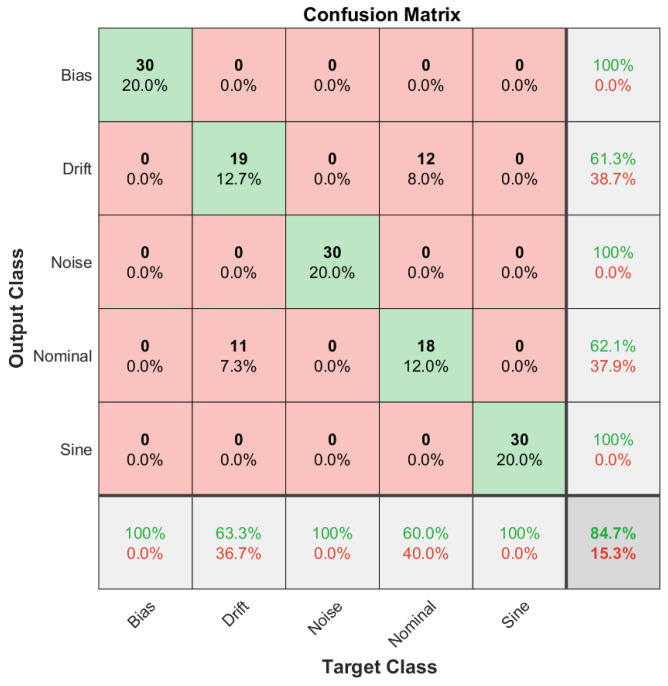
Confusion matrix for the test data set showing that a considerable amount of the misclassified samples are between drift fault and nominal scenarios.

The fault diagnosis accuracy is defined as the classification accuracy of the DCNN model for each data set corresponding to the train, validation and test data sets. Then, in order to visually inspect the classification accuracy of the DCNN model for each data set, we employed confusion matrices, which can be defined as a two-dimensional matrix with dimensions of true target classes and model output classes. In this configuration, the diagonal terms correspond to true classifications, where off-diagonal terms can be considered as misclassified samples. Confusion matrices are introduced for the training data set in Figure 12, for the validation data set in Figure 13 and for the test data set in Figure 14.

As it can be extracted from the confusion matrices, the overall accuracy of the methodology is limited by the case of the slow drift fault in the sensor reading. Other fault scenarios are successfully diagnosed for all training, validation and test data sets. However, misclassifications are high on the slow drift fault and nominal scenario cases. This property shows that the proposed methodology is successful for bias, noise and sine fault scenarios, but the diagnosis capability between the nominal and drift fault scenario is limited. The classification accuracy levels are presented in Table 2 for different cases of the reference model parameter $L^{\prime }$.

From Table 2, it can be stated that the sensor fault diagnosis framework performs well for the bias, noise and sine disturbance fault scenarios and for all training and validation sets as well as test sets. In addition, the procedure is applied for the different reference plant parameter $L^{\prime }$ for the robustness study. Overall, the performance is better in the system with almost no time delay where $L^{\prime }=0.1$, and a relatively lower performance is achieved for dramatic time delay where $L^{\prime }=10$. The robustness study, performed with the aid of reference system parameters, demonstrated that overall, the sensor fault diagnosis performance of the utilized methodology does not depend on a specific experimental condition. When the change in the reference plant parameter $L^{\prime }$ is considered, the methodology covers a broad range of applications. Moreover, the overall characteristics observed from the confusion matrices are still valid for different reference plant parameters. Among all the studied fault diagnosis scenarios, drift and nominal scenarios present a big proportion of the misclassified samples.

**Figure 15:**
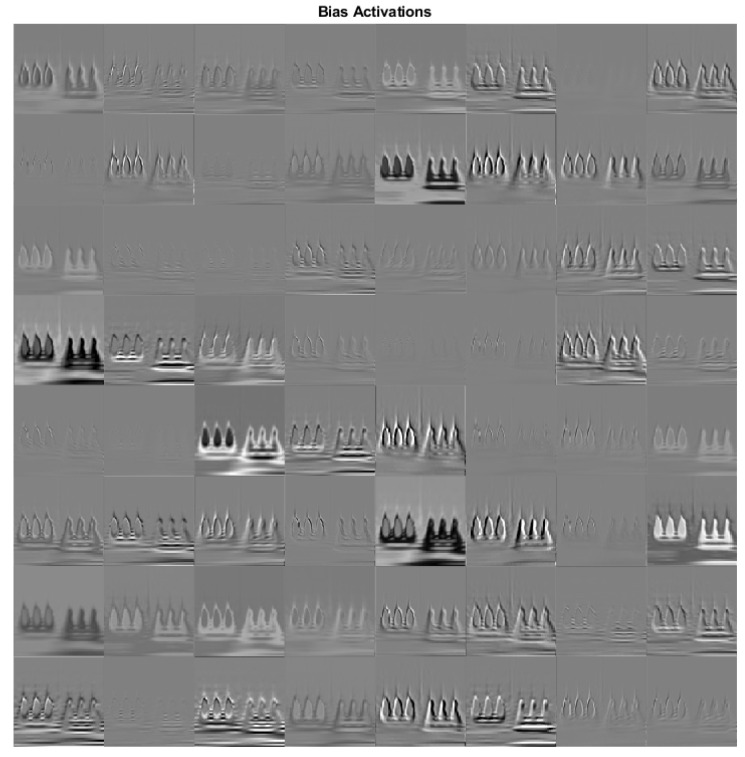
Sensor bias fault scenario-activated areas by convolutional layer.

To further examine the effect of the DCNN model, the activation areas on the original time-frequency representation images of data spatial temporal DMDc modes are utilized. A sample scenario for the sensor bias fault with activated areas by the convolutional layer are presented in Figure 15.

**Figure 16:**
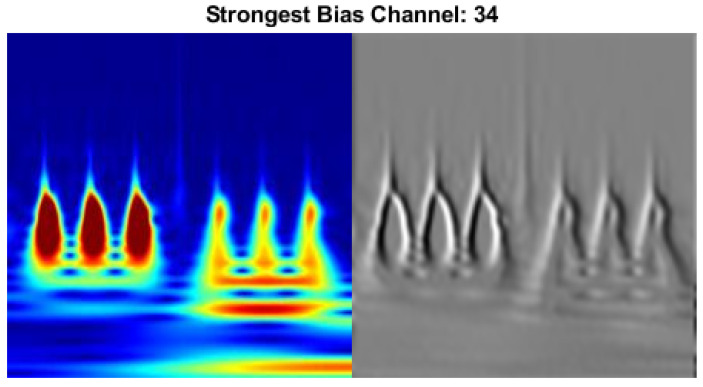
The strongest channel for sensor bias fault scenario.

In order to demonstrate the effectiveness of an activation on top of the original time-frequency representation of a sample scenario, the strongest activation channel is presented in Figure 16. The strongest channel refers to the feature image that provides information on the image in terms of the DCNN model.

**Figure 17:**
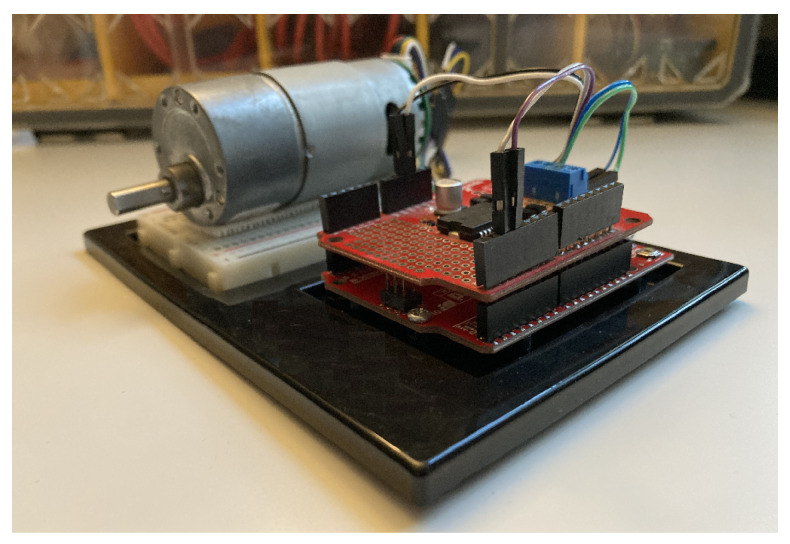
The target system as a velocity control in a real-time application.

To demonstrate the effectiveness of the framework, a target system is defined as a velocity control in a real-time application, as shown in Figure 17. The system consists of a ASLONG JGB37-545B 12V DC motor along with a Hall effect encoder YC-52010, Ardumoto Shield and Arduino Uno in hardware in the loop setup with a PID controller in MATLAB/Simulink. The proposed framework is applied to the velocity control system with a square reference signal with a period of 10 seconds, similar to the reference case study. Then, the sensor fault types defined in the reference case study are purposely injected into the hardware in the loop setup. After the methodology, the fault classification results for the real system yields to 78.7% training and 73.3% test accuracy. The results demonstrated that the framework generates successful fault diagnosis operation not only for a reference case study in simulations but also in a sample target system.

## 4. Conclusions

A systematic framework is presented in order to achieve sensor fault diagnosis using only the sensor measurements and reference input signals. The physics-informed transfer learning framework, first proposed in [37] for the fault cause assignment, is extended to investigate different sensor faults that can arise. Without the knowledge of the physics behind the system, a completely data-driven methodology, DMDc, enables one to extract the underlying dynamics. Then, the data spatial temporal modes obtained by DMDc transformed into time-frequency representation images using CWT to create the input layer of the DCNN model. Finally, a previously trained image classification algorithm, *GoogLeNet*, is implemented in order to perform transfer learning. By using the transfer learning methodology, the training effort is decreased dramatically while preserving the fault diagnosis accuracy. A reference case study with the FOPTD model is utilized in order to provide a benchmark sensor fault diagnosis application. An effective sensor fault diagnosis accuracy is achieved for all training, validation and test sets by the implementation of a systematic framework. Different plant parameters are implemented in order to perform robustness of the framework. High accuracy for the sensor fault diagnosis problem is demonstrated for bias, noise and sine fault scenarios. The confusion matrices presented within the results demonstrated that the diagnosis capability of the methodology can be improved for the drift fault scenario. Finally, a real-time application of the velocity control target system is utilized in order to demonstrate that the proposed methodology is valid for real sensor applications. There are no limitations on extending the proposed methodology for a range of different fault scenarios and even different fault severeness degrees. Once the corresponding labeled historical input–output data streams are available for a system, the methodology can offer a systematic way to perform fault diagnosis. Future work for the proposed methodology can be considered as an extension of the physics-informed transfer learning-based fault diagnosis framework into more complex real-world applications with labelled experimental data. 

## Figures and Tables

**Table 1:** DCNN model parameters.

Parameter	Value
Initial Learning Rate	0.0001
Mini Batch Size	30
Maximum Number of Epochs	1000
Minimum Gradient Magnitude	$1\;\times \;10^{-7}$

**Table 2:** Sensor fault diagnosis accuracy levels for each data set.

Set	$L^{\prime }=1$	$L^{\prime }=0.1$	$L^{\prime }=10$
Training	94.9%	91.5%	85.6%
Validation	90.0%	91.3%	76.7%
Test	84.7%	91.3%	80.7%

## Data Availability

The source codes for reproducibility that support the findings of this study are available from the GitHub link: https://github.com/fguc/SensorFDwithPITL, accessed on 7 March 2022.

## Abbreviations

The following abbreviations are used in this manuscript:

CWT	Continuous wavelet transform
DWT	Discrete wavelet transform
DCNN	Deep convolutional neural network
DMD	Dynamic mode decomposition
DMDc	Dynamic mode decomposition with control
FOPTD	First-order plant with time delay
ILSVRC	ImageNet Large-Scale Visual Recognition Challenge
PID	Proportional-Integral-Derivative
STFT	Short-Time Fourier Transform
SVD	Singular Value Decomposition
WVD	Wigner–Ville distribution
